# Bisphenol A Exposure Modifies the Vasoactive Response of the Middle Cerebral Artery

**DOI:** 10.3390/ijms26083896

**Published:** 2025-04-20

**Authors:** Henrique Eloi Costa, Margarida Lorigo, Elisa Cairrao

**Affiliations:** 1 CICS-UBI, Health Sciences Research Centre, University of Beira Interior, 6200-506 Covilhã, Portugal; eloi.costa@ubi.pt (H.E.C.); margarida.lorigo@gmail.com (M.L.); 2 RISE-Health, Department of Medical Sciences, Faculty of Health Sciences, University of Beira Interior, Av. Infante D. Henrique, 6200-506 Covilhã, Portugal; 3 FCS-UBI, Faculty of Health Sciences, University of Beira Interior, 6200-506 Covilhã, Portugal

**Keywords:** bisphenol A, endocrine disrupting compound, stroke, ischemia, middle cerebral artery, vasoconstriction, vasorelaxation

## Abstract

Bisphenol A (BPA) is the most used widely synthetic compound for the manufacture of polycarbonate plastics and epoxy resins produced worldwide. Given its androgenic and estrogenic activities, BPA is an endocrine disruptor that is linked to neurological and vascular outcomes, including strokes. Therefore, this study aims to investigate the mechanisms by which a 24 h exposure to BPA (0.002–20 μM) modifies the contractile function of rat middle cerebral artery (MCA) smooth muscle cells (SMCs). Thus, MCA explants were isolated from Wistar rats, and the SMC-MCA vasoactive response was assessed using planar cell surface area, while the gene expression of proteins and ion channel subunits involved in the MCA vasoactive response was evaluated by real-time quantitative PCR. The exposure to BPA (0.02 and 2 μM) decreased the noradrenaline (NA) vasocontractile response and sodium nitroprusside (SNP) vasorelaxant response. Moreover, exposure to BPA (0.02 and 2 μM) increased the gene expression of the soluble guanyl cyclase protein and the large conductance Ca^2+^-activated K^+^ channels (1.1 α-subunit). These results suggest an impairment of the SMC-MCA vasoactive response induced by intermediate BPA concentrations, an effect not attained for the lowest or highest exposure concentrations (non-monotonic inverted U-shaped response). In summary, these findings suggest that BPA exposure modifies MCA vascular homeostasis by interfering with the nitric oxide (NO) pathway and may, thus, be involved in ischemic stroke development.

## 1. Introduction

Nowadays, ecosystems and human health are both under threat from the manufacturing, usage, and waste of plastic. One of the most widely used synthetic compounds in the manufacture of polycarbonate plastics and epoxy resins produced around the world is bisphenol A (BPA) [1]. The production and use of BPA have increased progressively every year, with this endocrine-disrupting compound (EDC) being used in various types of materials, which has also expanded the number of routes of contamination, either through the use and wear of the containers in which it is found or through the pollution caused when they are disposed of in aquatic, marine, aerial and soil ecosystems [2]. This EDC is present in various products, such as toys, baby bottles, food containers, water pipes, medical and electronic equipment, thermal paper, and others [1,3]. However, since 2011, the European Union has increased restrictions on the use of this compound. Initially, it was banned in the manufacture of baby bottles, while in 2014 it was strictly limited to toys, and in 2024 it was banned in products that come into contact with food and drinks [4,5].

Due to BPA’s widespread use and endocrine-disrupting qualities, most notably estrogenic and androgenic, numerous studies have documented the compound’s effects on various human and animal physiological systems [6,7]. As a result, daily exposure to BPA has emerged as a serious public health concern, which led the European Food Safety Authority (EFSA) to further lower the tolerable daily intake value from 4 μg/kg bw/day set in 2015 to 0.2 ng/kg bw/day in April 2023 [8]. However, increased exposure to BPA does not always induce an increase in its effects, since BPA, as an EDC, may have a non-monotonic response (a typical U- or inverted U-shaped response). These characteristic responses imply a regulatory challenge when setting cutoff values for safe BPA exposure [9,10]. Furthermore, BPA has already been detected in biological matrices, such as blood, cell-free blood products, urine, saliva, breast milk, umbilical cord blood, and amniotic and semen fluids, as well as sweat and hair [11]. Moreover, in the brain, the accumulation of BPA has been reported. Geens et al. [12] reported BPA accumulation in the human brain samples at a concentration of 0.91 ng/g (0.00398 μM, considering that the density of brain tissue is approximately equal to the density of water), and Kim et al. [13] demonstrated BPA concentrations from 0.097 to 0.745 μg/g tissue in several specific brain tissues of female rats (0.000425 μM to 0.00326 μM, considering that the density of brain tissue is approximately equal to the density of water).

Though the impact of BPA on the brain system is still little understood, it is known that environmental exposure to BPA may have some effects on brain physiology and development. In this sense, BPA affects endogenous defense mechanisms and potentially requires exogenous interventions. These effects can, thus, be related to various neurological diseases, such as neurovascular diseases, neurodegenerative diseases, neurodevelopmental disorders, depression, emotional problems, anxiety, and cognitive disorders, among others [7,14].

Concerning the vascular effects of BPA, only a few studies have examined how BPA affected vasculature [15,16,17,18,19]. It has been shown that BPA can modulate ion channels by blocking L-type Ca^2+^ channels (LTCC) in A7r5 (a vascular smooth muscle cell line obtained from embryonic rat aorta cells) [15] and activating BK_Ca_ in coronary smooth muscle cells [16], leading to vasorelaxation. Moreover, BPA has also been shown to induce proliferation and apoptosis of the A7r5 and the human aorta [17]. On the other hand, BPA has also been shown to regulate blood pressure by inducing the uncoupling of angiotensin II/calcium/calmodulin-dependent protein kinase II-α from endothelial NO synthase [18]. Recently, BPA was associated with vascular dysfunction, altering vascular reactivity in human arteries, suggesting that it may induce cardiovascular pathologies [19]. Furthermore, Cai et al. [20] positively associated the prevalence of congestive heart failure, coronary heart disease, angina pectoris, myocardial infarction and stroke with urinary BPA. This study was carried out with US participants from the NHANES study between 2003 and 2014. Specifically, the association between BPA and stroke was more evident in men, among the 9139 participants, where 324 people suffered a stroke [20].

Stroke, also known as a brain attack, is the most common neurovascular disease, the second most common cause of death worldwide, and the third most common cause of permanent disability [21]. The most prevalent type of stroke is ischemic stroke, which is caused by ischemic occlusion, i.e., through the embolism or occlusion of large or small blood vessels, mostly in the middle cerebral artery (MCA) [22]. When cerebral blood flow to the brain is disrupted, brain cell death results, which is what causes a stroke. The MCA is responsible for irrigating the brain’s numerous lateral areas, and, depending on the regions that it affects, its impairment can cause a variety of symptoms [22,23]. Cerebral blood flow is sustained by the neurovascular unit, which is a functional and very complex unit made up of different types of cells [22]. These include smooth muscle cells (SMCs), which are vascular cells present in the tunica media of the cerebral blood vessels, and consequently in all the arteries and arterioles of the neurovascular unit, except in the cerebral capillaries, where pericytes are present [22,24]. SMCs are essential and multifunctional cells, showing more plasticity than any other cell type, and are organised in concentric layers around the basal lamina in order to acquire a variety of specialized skills [24,25]. They also regulate blood vessel diameter, blood pressure, and blood flow distribution, thus contributing to basal vascular tone [22]. However, SMCs also have various mechanisms by which they regulate both the vasodilation and vasoconstriction of cerebral blood vessels, i.e., through the phenomena of contraction and relaxation, allowing adequate blood pressure to be maintained [26].

The present work aims to investigate the mechanisms by which BPA modifies the vasoactive function of rats’ smooth muscle cells of the middle cerebral artery (SMC-MCAs). To achieve this, culturing of SMC-MCAs was performed to analyze these in vitro effects. The viability of the SMC-MCAs was tested by MTT assay in response to BPA. Moreover, to investigate the contractility response of SMC-MCAs after the conjoint application of two vasoactive agents, noradrenaline (NA), and sodium nitroprusside (SNP), a contractility assay using planar cell surface area (PCSA) was performed. Furthermore, real-time quantitative polymerase chain reaction (qPCR) tests were conducted to evaluate the gene expression of proteins and ion channel subunits implicated in the MCA contractile response.

## 2. Results

### 2.1. BPA Effects on Cell Viability (MTT Assays)

**Figure 1:**
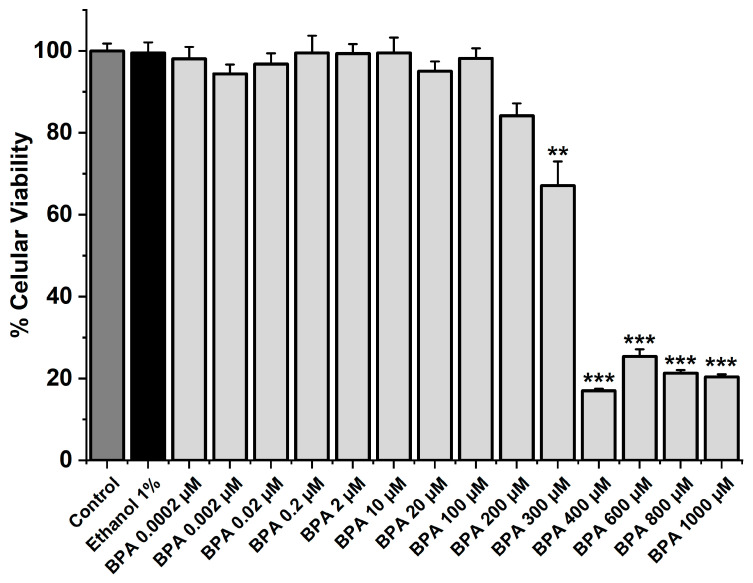
SMC-MCA viability percentage in response to 24 h exposure to BPA. A total of 6 MTT assays (from 6 rats, to minimize genetic variability) were performed with 4–8 replicates of each concentration. Cell viability is indicated as a percentage (%) in the data. Each bar represents the mean value, and the vertical line indicates the standard error of the mean (SEM). Statistical analysis was carried out using a Kruskal–Wallis one-way analysis of variance on ranks (*p* < 0.05), followed by multiple comparisons vs. vehicle group, ethanol 1% (Dunn’s method), where ** *p* < 0.01; *** *p* < 0.001. Abbreviations: smooth muscle cells of the middle cerebral artery (SMC-MCAs), bisphenol A (BPA).

The MTT assay was performed to analyze the cellular viability of SMC-MCAs exposed to BPA at different concentrations (0.0002, 0.002, 0.02, 0.2, 2, 10, 20, 100, 200, 300, 400, 600, 800, and 1000 μM). In addition to BPA, the cells were exposed to ethanol for 24 h, i.e., the solvent used to dissolve the BPA (vehicle), and to the culture medium as a control. Only the five highest BPA concentrations (300, 400, 600, 800, and 1000 μM) significantly reduced cell viability (*p* < 0.05), as Figure 1 illustrates. Comparing the other concentrations with the vehicle and the control, no changes were found. The original data can be seen in Table S1.

### 2.2. BPA Effects on Cellular Contractility

After obtaining the SMC cultures, the PCSA technique was used, based on what had been previously established by our research group. In this way, the vasoactive response of SMC-MCAs, previously incubated (for 24 h) with different concentrations of BPA (0.002, 0.02, 0.2, 2, and 20 μM), to 1 µM of the contractile agent NA and 1 µM of the relaxing agent SNP was evaluated.

**Figure 2:**
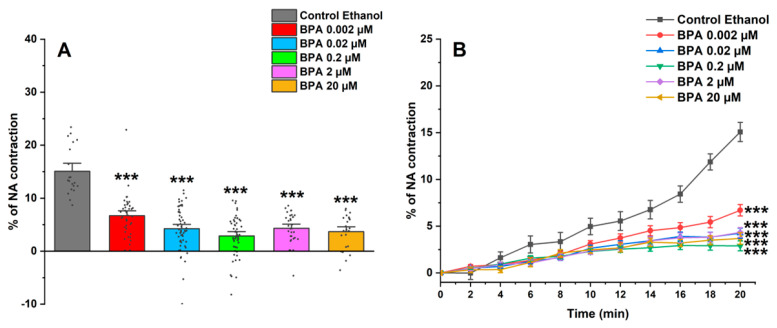
(**A**) Effect of the action of 1 μM of NA on SMC-MCAs. Percentage of NA-induced contraction after 24 h of incubation with exposure to various concentrations of BPA (0.002–20 μM). A total of 6 experiments (from 6 rats, to minimize genetic variability) were conducted, and all concentrations were tested in triplicate for each primary cell culture. Data are expressed as percentage (%) of contraction of noradrenaline over the basal area. The bars represent the mean ± standard error of the mean (SEM), and the small black dots represent the values. Statistical analysis was carried out using a Kruskal–Wallis one-way analysis of variance on ranks (*p* < 0.05), followed by Dunn’s method, where *** *p* < 0.001. (**B**) Temporal profile of the contraction induced by the action of NA (1 μM) in the first 20 experimental minutes, every 2 min, in SMC-MCAs exposed for 24 h with various BPA concentrations (0.002–20 μM). Statistical analysis was carried out using the two-way ANOVA method, followed by multiple comparisons vs. the control group (Holm–Sidak method), where *** *p* < 0.001. Abbreviations: middle cerebral artery smooth muscle cells (SMC-MCAs); bisphenol A (BPA); noradrenaline (NA).

Figure 2A shows the maximum effect induced by NA after incubating SMC-MCAs in BPA and ethanol (0.01%) as a control; this effect was obtained 20 min after the addition of NA, the time needed for maximum contraction to occur. Furthermore, the time profile of SMC-MCA contraction with NA was also demonstrated in Figure 2B. Overall, the BPA exposure induced a decrease in the NA contractile response when compared with the control.

**Figure 3:**
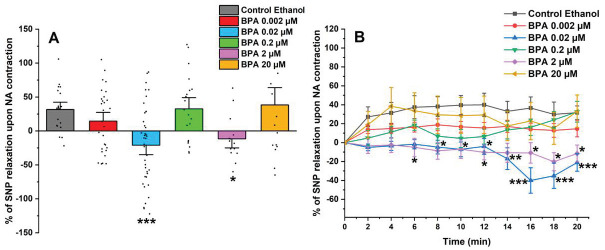
(**A**) Effect of the SNP relaxation (1 μM) on SMC-MCAs after contraction with 1 μM of NA. Percentage of relaxation induced by SNP after 24 h of incubation with exposure to various concentrations of BPA (0.002–20 μM). A total of 6 experiments (from 6 rats, to minimize genetic variability) were conducted and all concentrations were tested in triplicate for each primary cell culture. The data are expressed as a percentage (%) of relaxation over the basal area, upon contraction with noradrenaline. The bars represent the mean ± standard error of the mean (SEM), and the small black dots represent the values. Statistical analysis was carried out using a Kruskal–Wallis one-way analysis of variance on ranks (*p* < 0.05), followed by Dunn’s method, where * *p* < 0.05 and *** *p* < 0.001. (**B**) Temporal profile of SNP (1 μM) relaxation, after contraction with NA (1 μM), every 2 min, in SMC-MCAs incubated for 24 h with various concentrations of BPA (0.002–20 μM). Statistical analysis was carried out using the two-way ANOVA method, followed by multiple comparisons vs. the control group (Holm–Sidak method), where * *p* < 0.05, ** *p* < 0.01 and *** *p* < 0.001. Abbreviations: middle cerebral artery smooth muscle cells (SMC-MCAs); bisphenol A (BPA); noradrenaline (NA); sodium nitroprusside (SNP).

After exposing the SMC-MCAs to the contractile agent, the vasoactive effect of 1 µM of SNP was analyzed (shown in Figure 3A). The SNP induced a relaxant effect in SMCs exposed to the control (ethanol 0.01%) and 0.002, 0.2, and 20 μM of BPA. However, after 0.02 and 0.2 μM of BPA exposure, the SMC-MCAs underwent a contraction in response to SNP, contrary to the observed for the control. Figure 3B represents the percentage of SNP relaxation upon NA contraction over time, with the maximum contraction induced by NA considered to be zero. The statistical analysis of Figure 3B was shown in Table 1.

**Figure 4:**
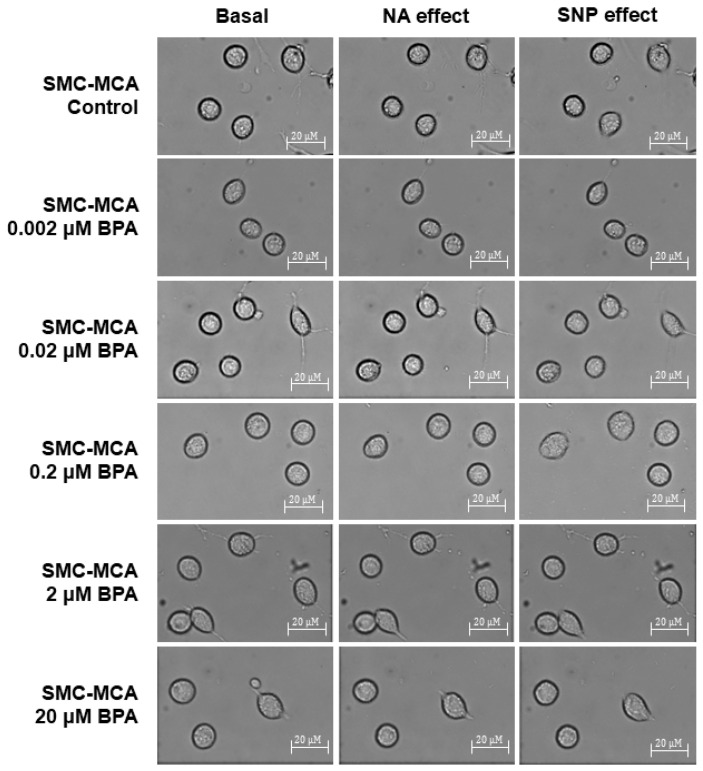
Sample photos from a typical SMC-MCA contractility experiment captured through the planar cell surface area (PCSA) technique. The basal column represents the cells without vasoactive agent addiction, the effects of noradrenaline are shown in the NA column (where a decrease in cell area can be seen, which corresponds to a vasoconstrictor effect), and the effects of sodium nitroprusside are shown in the SNP column (where an increase in cell area can be seen, which corresponds to a vasorelaxant effect), after a 24 h pre-incubation period with various concentrations of BPA (0.002–20 μM). Abbreviations: middle cerebral artery smooth muscle cells (SMC-MCAs); bisphenol A (BPA).

Furthermore, Figure 4 shows representative images of the SMC contractility experiments. Briefly, in each experiment, a microscope field-of-view with three or more cells is chosen. The area of these cells is measured at the basal time (20 min without exposure to any vasoactive agent). Subsequently, NA (the contractile agent) is added, and the area of the previously selected cells is measured every 2 min for 20 min. After this contraction, SNP (the vasorelaxant agent) is added, and the same area measurement procedure is performed. This procedure was conducted to control cultures and those exposed for 24 h to various concentrations of BPA (0.002–20 μM). The procedures to perform PCSA in the SMC-MCAs were optimized in articles previously published by our group [27,28]. The control experiments with the solvent were performed, and no variations were obtained for the cell area after the basal time. Moreover, the data of the baseline are similar between the samples with and without BPA incubation.

### 2.3. BPA Effects on the Expression of Genes Implicated in the Contractile Properties of SMC-MCAs

**Figure 5:**
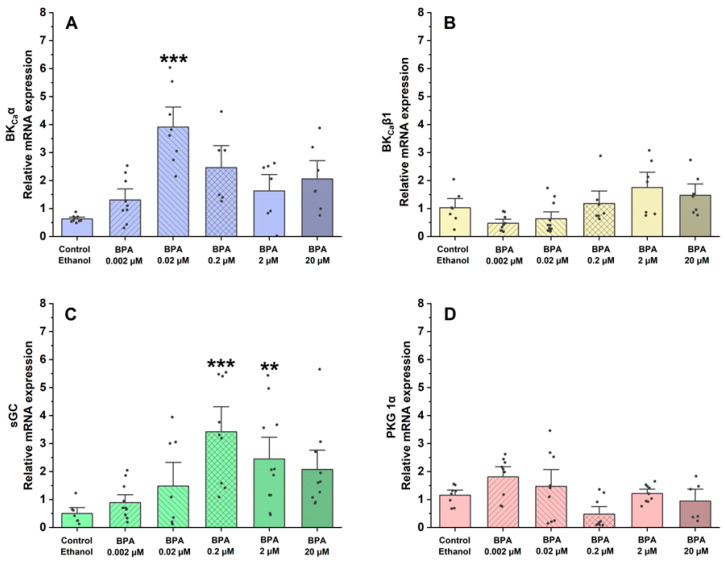
Relative mRNA expression in SMC-MCAs exposed to various BPA concentrations (0.002–20 μM): (**A**) BK_Ca_ 1.1α-subunit; (**B**) BK_Ca_ β1-subunit; (**C**) sGC; (**D**) PKG 1-α. Cyc A was the housekeeping gene that was utilized to normalize mRNA expression. A total of 6 experiments (from 6 rats, to minimize genetic variability) were conducted, and all concentrations were tested in triplicate for each qPCR. The bars represent the mean ± standard error of the mean (SEM), and the small black dots represent the values. Statistical analysis was carried out using one-way ANOVA (*p* < 0.05), followed by Dunnett’s post hoc test, where ** *p* < 0.01 and *** *p* < 0.001. Abbreviations: smooth muscle cells of the middle cerebral artery (SMC-MCAs); bisphenol A (BPA).

Using qPCR, the assessment focused on the gene expression of proteins and ion channels that are involved in controlling vascular tone and the regulation of SMC contraction and relaxation mechanisms. In this sense, the effects of BPA on the mRNA expression of BK_Ca_ 1.1α (Figure 5A) and β1-subunits (Figure 5B) and proteins implicated in the contractile process of SMC-MCAs, sGC (Figure 5C), and PKG 1α (Figure 5D) were examined.

Regarding the relative mRNA expression of the BK_Ca_ 1.1 α-subunit channels, compared to the control (ethanol), the results showed that it was significantly higher in SMC-MCAs exposed to 0.02 μM (*p* < 0.001 of BPA). Conversely, in the BK_Ca_ β1-subunit channels, at all concentrations of BPA, the effects were similar to the control. Concerning sGC protein expression, relative mRNA expression was significantly higher at concentrations of 0.2 μM (*p* < 0.001) and 2 μM (*p* < 0.01), although no statistical differences were observed for the relative PKG mRNA expression.

## 3. Discussion

In this research, the BPA effects on the modulation of the vasoactive response of SMCs derived from rat MCA were analyzed. The BPA exposure was assessed using several concentrations chosen according to the physiological and environmental concentrations [9,29,30]. BPA has been detected in human urine samples (31.9 μg/L, corresponding to 0.1397 μM) [9], in human brain samples (0.91 ng/g, corresponding to 0.00398 μM) [12], and between 0.097 to 0.745 μg/g tissue (0.000425 μM to 0.00326 μM) in specific rat brain tissues [13]. In surface water and groundwater environmental samples, Cruz-López et al. [30] found BPA concentrations between 20 and 25 μg/L (0.0876–0.1095 μM). Moreover, the chosen concentrations attended to the in vitro–in vivo scaling factor [31]. This factor can be described as the requirement for in vitro concentrations 20–200 times higher than the highest concentration in human plasma to have comparable biological effects. Thus, considering the C_max_ (18.9 ng/mL, corresponding to 0.0828 μM) of BPA in human plasma [19], and the physiological and environmental concentrations, the concentrations used in this study ranged from 0.0002 to 20 µM (approximately 200× of C_max_).

Firstly, the MTT assay was conducted to determine the cell viability of SMC-MCAs in response to exposure to fourteen distinct concentrations of BPA. Compared to the negative control, only the five highest concentrations, namely 300, 400, 600, 800, and 1000 μM, significantly reduced the viability by approximately 33, 83, 72, 79, and 80%, respectively, suggesting that BPA has a toxic effect on SMC-MCAs. Therefore, the BPA concentrations used in the subsequent studies (0.002, 0.02, 0.2, 2, and 20 μM) did not result in cell toxicity.

The vasoactive response of SMC-MCAs was then assessed using the PCSA technique in response to NA, as a contractile agent, and SNP, as a relaxing agent. The two agents were chosen based on research previously conducted on this type of cell, which revealed changes in the vasoactive response when these two agents were administered [27,28]. In the cerebral arteries, and, consequently, in the SMC-MCAs, there is an abundant expression of α1-adrenergic receptors, which are activated in the presence of NA, as it is an agonist of these receptors [32]. The connection between NA and α1-adrenergic receptors, due to the Gq proteins that are coupled to these receptors, promotes an increase in the activity of phospholipase C (PLC), causing the hydrolysis of phosphatidylinositol biphosphate and the production of inositol 1,4,5-triphosphate (IP3) and diacylglycerol (DAG). These two molecules act as second messengers to facilitate the release of calcium (Ca^2+^) from intracellular reservoirs and activate protein kinase C (PKC), which triggers signaling cascades necessary for the contractile process, thus causing SMC vasoconstriction [33,34]. However, NA does not only have a vasoconstrictive effect, as cerebral arteries also express β1 adrenergic receptors, which cause relaxation through processes related to potassium (K^+^) channels when activated [34]. These receptors are linked to Gs proteins that activate adenylate cyclase, which in turn regulates vasorelaxation [35]. Thus, the results obtained are in agreement with the previously mentioned studies, i.e., after the addition of NA, all SMCs exposed to BPA showed contraction, although less than the control, especially at exposure concentrations of 0.02, 0.2, 2, and 20 μM of BPA. Under these conditions, the SMCs may be compromised in terms of α1-adrenergic receptor activity, since the contractile response was more significantly reduced the higher the concentration of BPA that the cells were exposed to.

On the other hand, the vasorelaxant response of SMC-MCAs has also been evaluated, since these cells can contract and relax to maintain blood flow and respond to different stimuli effectively [22]. The vasodilator agent, SNP, is a nitric oxide (NO) donor that promotes the activation of sGC by increasing its intracellular concentration, thus causing an increase in cyclic GMP (cGMP) levels [36,37,38], leading to PKG activation. Through the activation of PKG, BK_Ca_ channels and voltage-sensitive K^+^ channels (Kv) are activated and LTCCs are inhibited. As a result, the amount of intracellular Ca^2+^ decreases, due to the activation of K^+^ channels [39]. According to our findings, in response to SNP as a vasodilating agent, SMC-MCAs exposed to BPA showed concentration-dependent effects. The vasodilator responses of SNP were always observed, as expected, compared to the control, except for cells incubated with 0.02 and 2 μM of BPA. In these cases, it was possible to observe an opposite response, i.e., a vasocontractile response. These results suggest that the exposure of SMC-MCA to BPA can interfere with the NO and sGC pathways by modulating sGC, which is in line with previous investigations in the human umbilical artery [19] and uterine arteries [40]. Fonseca et al. [19] observed that the chronic exposure of BPA at higher concentrations decreased the activity of the NO/sGC/cGMP/PKG pathway, inducing a lower vasorelaxant response when the arteries were contracted with serotonin and KCl, and that this decrease in vasorelaxation is dependent on the exposure concentration, as was observed in this study. In agreement with Fonseca et al. [19], our data seem to suggest a compensatory or negative feedback mechanism, where a decrease in activity promotes an increase in sGC mRNA expression in an attempt to increase activity (homeostasis) [41]—a role also played by sex hormones [9]. The sGC is the main enzyme that mediates the biological actions of NO, which promotes an increase in the cellular concentration of cGMP, which promotes the activation of PKG and activates BK_Ca_, and is one of the main mechanisms responsible for vasodilation at arterial level [19]. In agreement with Fonseca et al. [19], sGC mRNA expression was significantly higher for 0.2 and 2 μM of BPA. On the contrary, an increase in the relative mRNA expression of the α subunit channels of BK_Ca_ 1.1 was observed in SMC-MCAs exposed to 0.02 μM of BPA, while Fonseca et al. [19] obtained gene expression changes for the β1 subunit of BK_Ca_ 1.1. From a structural and functional point of view, BK_Ca_ channels exist as tetramers of α subunits forming the ion channel pore together with regulatory β subunits. The tissue-specific β1 subunit, which is linked to the α subunit, increases the Ca^2+^ sensitivity of these channels, allowing vascular smooth muscle to relax [42]. These structural alterations may be the basis of the different effects found between the two studies, carried out on different vascular models. However, previous studies have shown that BPA activates BK_Ca_ [16] mainly due to the β1 subunit [43]. In cultures of rat hippocampal neurons, it has already been shown that incubation of BPA concentrations below 0.1 μM rapidly activates the Ca^2+^ signaling system through activation of non-genomic pathways, including estrogen receptor (ER) α and β [44], and this was also observed by Zhong et al. [45] at concentrations below 10 μM. Furthermore, in a study with human embryonic stem cells, at concentrations below 10 μM, BPA caused an excessive influx of Ca^2+^ [46], which corroborates our data in which there was a decrease in the relaxant response induced by SNP, with contraction occurring even at intermediate concentrations of BPA. Thus, based on these findings, it is clear that BPA affects the expression of the sGC protein and the α 1.1 subunit channels of BK_Ca_, which may be the explanation for the results obtained in PCSA. Therefore, the expression studies supported vascular activity experiments, the focus of this research. It should be noted that mRNA does not always reflect the level of proteins and that various transcription control mechanisms (e.g., post-translational modifications) can alter protein quantity and function. Even when present, the activity of a protein is solely responsible for the physiological response (not the quantity), since there can be low quantities of protein with high activity (or vice versa) [47,48]. Moreover, the SMCs have a compartmentalized signaling of cyclic nucleotides, which is mainly controlled by phosphodiasterases—crucial for activating various ion channels involved in vascular contractile mechanisms [9,49]. In this sense, an interesting strategy to be applied in future studies would be the realization of Western blot studies or immunoenzymatic assays (e.g., ELISA) focused on protein analysis within the compartmentation scope. Additionally, transcriptome-wide mRNA profiling may also represent a valuable future approach to identify other molecular targets or signaling pathways affected by BPA exposure, thereby complementing the functional findings of the present study.

Indeed, rodent models have been widely used in preclinical studies at the human cardiovascular level [50]. Despite this, some limitations must be considered when extrapolating results. For example, the use of in vitro models, as in this study, in which cells were isolated from MCA explants, may not fully reflect the complexity of in vivo conditions, including the interactions between different cells [19]. In this study, the MCA cultures carried out were previously characterized as having around 90% SMCs and 10% endothelial cells (ECs) [27]. In this sense, the MCA cultures carried out for SMCs were considered pure. Considering the vascular role of ECs in the regulation of vascular contractility by SMCs, namely through the release of local vasodilator and vasoconstrictor mediators, it is important to consider the close correlation between both types of cells [9]. However, in this study this correlation could not be analyzed, given that the type of explant culture was optimized to obtain SMCs. Despite this, it would be interesting in the future to optimize a new method for culturing MCA endothelial cells or co-culturing both [51]. At the vascular level, there may be differences between EDCs and SMCs, which should be considered (e.g., sex-specific effects). Indeed, gender differences for vascular ECs have already been reported, while for SMCs there appear to be no differences in the effects of EDCs [9]. Therefore, it is expected that the effect of BPA is not sex-specific in SMCs at the brain level, but future studies would be necessary to prove this hypothesis. This issue also highlights the need to explore the effect of BPA at the smooth muscle and endothelial level, considering its possible effects on fetal programming, according to the DOHaD theory [9,52]. Another important issue is the differential role of sex hormones in the gender-specific brain. For example, the administration of a single intranasal dose of 17-β-estradiol in healthy postmenopausal women increased cerebral perfusions, while the effect on peripheral circulation was much more limited [53], and differences in ophthalmic artery perfusion were also observed [54]. In this research, only male Wistar rats were used, so the action of BPA on the effects of estradiol was not directly analyzed. It would, therefore, be interesting in future studies to carry out a direct or comparative study in female rats to clarify the estrogenic action of sex-specific BPA at brain level. On the other hand, the exposure time to BPA (24 h) should also be a factor to consider in chronic studies in future in vivo investigations. Indeed, in in vitro studies, such as this research, or ex vivo [19], 24 h of exposure is a sufficient time to demonstrate changes in gene expression and vascular reactivity. To carry out contractility studies, SMCs must express a contractile phenotype which is only guaranteed up to 48 h of exposure (after this time, the SMCs lose their viability). Therefore, if the exposure time is extended in in vitro studies, consideration should be given to carrying out studies focused on phenotypic modulation involving SMCs in a contractile and synthetic state. For the long-term studies (in vivo studies, in these cases), longer exposure times should be considered to obtain more robust data.

In summary, these data indicate that BPA modifies the vasoactive response of SMC-MCAs, decreasing the vasocontractile response of NA and promoting a decrease in the vasorelaxant response of SNP upon chronic incubation with BPA in rat cerebral arteries. These results showed that the pathways involved in these responses promote altered gene expression of BK_Ca_ 1.1 α-subunit channels and sGC. Therefore, these data seem to indicate that BPA promotes the alteration of cerebral vasoreactivity through the NO and sGC pathway, which seems to be modulating the BK_Ca_ channels response. Indeed, many studies have linked altered NO levels and excessive Ca^2+^ influx in various areas of the brain. On the other hand, it was possible to observe that moderate concentrations of BPA (in terms of the action of SNP and the expression of BK_Ca_ 1.1 α-subunit channels and sGC) have a higher effect on SMC-MCAs compared to low and high concentrations, similar to the results observed for the ethanol control. This phenomenon, typical of EDC response, is characteristic of the non-monotonic inverted U-shaped curve of the BPA effects, as mentioned above, in which higher and also lower doses may not necessarily cause greater damage to the cells under study [10,55,56]. Nevertheless, additional research is required to fully understand its role and the related mechanisms. It is essential to investigate how and through which processes EDCs, such as BPA, can interfere with the normal physiological regulation of SMC-MCAs, since we are constantly interacting with products that contain them. This knowledge will help us to treat and prevent neurovascular diseases, such as ischemic stroke. Given the decrease in the NO/sGC/cGMP/PKG signaling pathway induced by BPA, it is important to note that the dysregulation of NO levels may be implicated in endothelial dysfunction. This multifaceted pathological condition is considered an intermediate phenotype of unpredictable cardiovascular disease. Furthermore, endothelial dysfunction may not only represent a marker of vascular disease but also play an important pathogenic role, leading to disease progression and unfavorable outcomes, including stroke [57].

## 4. Materials and Methods

### 4.1. Smooth Muscle Cell Culture Through the Isolation of the Middle Cerebral Artery

The procedure used to obtain SMCs was based on the model created by our research group [27] from the MCA of male Wistar rats maintained at Direção-Geral de Alimentação e Veterinária (DGAV)-accredited CICS-UBI animal facilities. Twenty-one rats were employed in this method.

All experiments followed the European Convention for Protection of Vertebrate Animals Used for Experimental and Other Scientific Purposes (Directive 2010/63/EU) and were approved by the UBI Animal Welfare Body (ORBEA, Orgão de Bem-Estar e Ética Animal; approval Code: T0023, 22 March 2022).

Briefly, the Wistar males were anesthetized with ketamine (87.5 mg/kg) and xylazine (12.5 mg/kg) and then decapitated. The brain was removed with the proper surgical tools and then placed in a cold phosphate-buffered saline solution (PBS). The PBS included the following contents (mM): KCl (2.7), Na_2_HPO (10), KH_2_PO_4_ (2), and NaCl (137), with a pH of 7.4. Following their explantation, both MCAs were put in a well of a culture plate that had been pre-coated with collagen (5 μg/cm^2^). After a 3 min incubation period at 37 °C in 95% air and 5% CO_2_, 1 mL of the complete culture medium was added. With a pH of 7.4, the culture medium consisted of Dulbecco’s modified Eagle’s medium/nutrient mixture F-12 Hams (DMEM-F12, D8900-10X1L; Sigma-Aldrich, Lisboa, Portugal) supplemented with L-ascorbic acid (20 mg/L; Fisher Chemical, Fisher Scientific, Lisboa, Portugal), NaHCO_3_ (1.2 g/L; Labkem, Lisboa, Portugal), heat-inactivated fetal bovine serum (FBS; 5%; Gibco, Lisboa, Portugal), bovine serum albumin (BSA, 0.25%; Fisher Chemical, Fisher Scientific), insulin (5 μg/mL; Sigma-Aldrich, Portugal), heparin (2 μg/mL; Panreac Química, Lisboa, Portugal), fibroblast growth factor (FGF, 0.5 ng/mL; Sigma-Aldrich, Lisboa, Portugal), and epidermal growth factor (EGF, 5 μg/mL; Sigma-Aldrich, Lisboa, Portugal), plus a combination of streptomycin (10 mg/mL; PAN Biotech, Lisboa, Portugal), penicillin (10,000 U/mL; PAN Biotech, Lisboa, Portugal), and amphotericin B (25 μg/mL; PAN Biotech, Lisboa, Portugal). Additionally, 1 mL of a mixture of antibiotics was added to 100 mL of the culture medium. Moreover, 1 mL of culture medium was added after 24 h, during which the explants were kept in a humidified atmosphere with 5% CO_2_ at 37 °C. Thereafter, the culture medium was changed every 2 days and, after 20–25 days, confluent primary cultures (80–90% ratio) were obtained by the 3rd or 4th passages. These primary cultures were used for future assays after incubation with BPA (as detailed in the following sections). The BPA concentrations were chosen based on physiological (in human brain samples [12] and specific rat brain tissues [13]) and environmental [9,29,30] reported concentrations. In this procedure, the SMCs were maintained in DMEM F-12 (control) or with BPA (experiments).

### 4.2. MTT Assays

The MTT assay assessed the SMC-MCA viability after BPA exposure. The procedure was performed according to the manufacturer’s instructions (Sigma-Aldrich). Briefly, the confluent cells in 96-well plates were placed in a serum-free medium (DMEM-F12, BSA 0.25%; pH = 7.4) and incubated for 24 h with fourteen different concentrations of BPA (0.0002, 0.002, 0.02, 0.2, 2, 10, 20, 100, 200, 300, 400, 600, 800, and 1000 μM), dissolved in serum-free medium. At the end of the incubation period, the medium was replaced with 100 μL of culture medium containing MTT solution (3-[4,5-dimethylthiazol-2-yl]-2,5-diphenyltetrazolium bromide; 0.5 mg/mL). The solution was removed after 4 h of incubation at 37 °C, 5% CO_2_, and 95% humidity. When dissolved in DMSO (100 μL), the formazan crystals became purple to represent the amount of formazan formation. A photometer (EZ Read 400, Microplate Reader, Biochrom, Lisboa, Portugal) was used to measure the colour intensity at 570 nm.

### 4.3. Contractility Investigation Through Planar Cell Surface Area (PCSA)

According to earlier research that our group published, studies on cell contractility using the PCSA approach were conducted [27,28]. With this method, cell contractility can be analyzed in relation to variations in the cell surface area.

After reaching confluence, SMCs were placed in the serum-free medium for 24 h. Subsequently, a 24 h incubation period with five BPA concentrations dissolved in serum-free medium (0.002, 0.02, 0.2, 2, and 20 μM) was performed. These cells’ exposure for 48 h in a serum-free medium, after confluence, allowed them to switch from the synthetic phenotype to the contractile phenotype (the typical phenotype of SMCs when present in blood vessels). Moreover, the incubation period (24 h) with BPA for the SMC-MCAs was chosen according to previous studies performed by our research group [19], which confirmed that 24 h was sufficient for genetic alterations to occur. These data indicate that within a few hours of exposure, EDCs start to have long-term impacts on vascular SMCs, resulting in modifications in gene expression regulation, which can be reflected in alterations in the arteries’ contractile characteristics.

Posteriorly, the SMC-MCAs were trypsinized using a trypsin (0.3%) solution in a Ca^2+^-Mg^2+^-free, phosphate-buffered solution with EDTA (0.025%). The cells were plated (500 µL) in specific Petri dishes that had been previously coated with collagen (5 mg/cm^2^). The plates were put in an incubator for four hours, with an atmosphere of 95% air and 5% CO_2_ at 37 °C. Following this incubation time, the cells were washed four times using Krebs bicarbonate solution (495 µL), previously gassed with carbogen at 37 °C, and then examined under an inverted fluorescence microscope. The composition of the Krebs solution was as follows (mM): NaCl (119), KCl (5), CaCl_2_·2H_2_O (1.5), MgSO_4_·7H_2_O (1.2), KH_2_PO_4_ (1.2), NaHCO_3_ (25), EDTA-Na_2_ (0.03), l-( +)-ascorbic acid (0.6), and glucose (11) at a pH of 7.4.

To observe the cells, an inverted fluorescence microscope (Zeiss Axio Observer Z1, Jena, Germany) was used, characterized by being fully motorized; it consisted of a high-speed monochrome digital camera Axio Cam Hsm (Zeiss, Jena, Germany) and a built-in incubation system allowing temperature control (at 37 °C), which is crucial for maintaining the viability of the cells during the experimental process. Through the program AxioVision 4.8 (Zeiss, Jena, Germany), the supplementary “Automatic Measurement program” tool was used to analyze the cell surface area.

Subsequently, two distinct vasoactive drugs were used to assess the SMC vasoactive response, namely 1 µM of noradrenaline (NA) and 1 µM of sodium nitroprusside (SNP). These vasoactive agents were chosen to allow the analysis of the vascular dynamics by vasoconstriction and vasodilatory mechanisms. The experiments had a duration of 1 h; the first 20 min were spent as the basal phase, letting the cells rest; after that, the NA was added, and, after 20 min, it reached its maximal effect (maximum % contraction induced by NA); the final 20 min were then spent with the SNP acting as a vasodilatory agent. The choice of twenty minutes for this technique is explained by the fact that this is the time needed to obtain a maximum response in which the plateau phase of the effect is achieved. The concentrations of NA and SNP were based on previous investigations by our research group [27,28] using these cells (rat SMC-MCAs) and the same contractility protocol. These concentrations (NA 1 µM and SNP 1 µM) correspond to the supramaximal concentrations of these agents to induce the maximum response. Throughout each experiment, microphotographs were taken (every 2 min) of a minimum of three cells in order to obtain a graph of the temporal response of all the vasoactive agents added to the cells. However, cells with an abnormal morphology or that had died were excluded. Furthermore, for the cell area measurement, the aggregated cells were not considered for the data. The images obtained from the experiments were then analyzed and processed, and the difference in cell area after the addition of the vasoactive agents was measured.

### 4.4. The qPCR Analysis

#### 4.4.1. mRNA Extraction

The SMC-MCAs were subjected to various BPA concentrations (0.002–20 μM) for 24 h, and RNA was extracted according to the manufacturer’s instructions with some modifications. The quality of the extracted RNA was confirmed by 1% agarose gel electrophoresis, and, through the determination of the absorbance ratio at 260/280 nm, the purity of isolated RNA was verified. Moreover, the quantification of the RNA extracted (μg/μL) was determined via spectrophotometry with a nanophotometer (Implen GmbH, Munich, Germany).

#### 4.4.2. cDNA Synthesis

Regarding the synthesis of cDNA, extracted total RNA (1 μg) was used. At first, at 65 °C in a reaction, RNA was denatured for 5 min with Random hexamer mix (MB12901, NZYTech, Lisboa, Portugal) and dNPTs NZYMix (MB08601, 25 mM each, NZYTech, Portugal). Afterwards, NZY M-MuLV Reverse Transcriptase (MB08301, 20,000 U, NZYTech, Portugal) and reaction buffer (10×) were added, attaining a final volume of 20 μL. Firstly, the RNA was reverse-transcribed using random-hexamer primers incubated at 25 °C for 10 min and then at 37 °C for 1 h. In the final procedure, the reaction was inactivated by heating at 70 °C for 15 min, according to the manufacturer’s instructions (NZYTech, Lisboa, Portugal). All these steps were performed using a thermocycler (T100TH Thermal Cycler, BioRad, Hercules, CA, USA).

#### 4.4.3. Quantification of mRNA Expression by qPCR

The qPCR was employed to evaluate the mRNA expression of a few proteins and ion channel subunits implicated in the vascular vasoactive response, according to our earlier works [19,41]. For this purpose, to investigate the response to BPA treatment, the mRNA levels of distinct genes (large-conductance Ca^2+^-dependent K^+^ channels (BK_Ca_) subunits -α 1.1 and β1, soluble guanylate cyclase (sGC) and protein kinase cGMP-dependent 1 α-subunit (PKG 1α)) were accessed (Table 2).

For each qPCR reaction, NZYSupreme qPCR Green Master Mix (2×) (MB41903, NZYTech, Portugal) and 1 μL of generated cDNA (final volume of 20 μL) were used. The Cyclophilin A (Cyc A) gene was utilized to standardize the gene expression levels for each qPCR. The experiments were then carried out on the CFX Connect^TM^ Real-Time System (BioRad, Hercules, CA, USA), where primer optimization was carried out as described by Mariana et al. [41], including amplification efficiency, concentration (300 nM), annealing temperature, and cycling conditions. All samples were examined in triplicate. The standard mathematical model was utilized to calculate the relative mRNA expression, with the formula 2^−ΔΔCt^ [58].

### 4.5. Drugs and Chemicals

Regarding the drugs and chemicals utilized in this investigation, bisphenol A (BPA), noradrenaline (NA), sodium nitroprusside (SNP), Na_2_HPO_4_, absolute ethanol, DMEM-F12, Na_2_HPO, insulin, FGF, EGF, EDTA-Na_2_,and glucose were obtained from Sigma-Aldrich Química (Sintra, Portugal). Fluka, Honeywell provided the NaCl and KH_2_PO_4_; Panreac Quimica provided the heparin, CaCl_2_, and KCl; Labkem provided the MgCl_2_ and MgSO_4_·7H_2_O; and NaHCO_3_, BSA, glucose and l-(+)-ascorbic acid were acquired from Fisher Chemical, Fisher Scientific. Furthermore, FBS was acquired from Gibco and the antibiotic solution was acquired from PAN Biotech.

All the reagents used in qPCR techniques were purchased from NZYTech (Lisboa, Portugal), except the Tri reagent, which was from GRiSP (Porto, Portugal).

Absolute ethanol (99.8%) was used to dissolve BPA (stock solution-0.1 M), whereas distilled water was used to dissolve stock solutions of SNP (stock solution-0.01 M) and NA (stock solution-0.01 M). Every solution was kept in storage at −20 °C. It was necessary to prepare dilutions for the working solutions used in the different methodologies always on the day of the experiment. Controls in contractility and gene expression experiments were implemented by using ethanol in a maximum concentration which never exceeded 0.1%. In the MTT assay, the maximum ethanol concentration was 1%, corresponding to the maximum BPA concentration.

### 4.6. Statistical Analysis

The mean ± standard error of the mean (SEM) of n independent experiments was used to express the results. The PCSA contractility data were expressed for (1) NA contraction as percentage of contraction of noradrenaline over the basal area and (2) SNP relaxation as percentage of relaxation over the basal area, upon contraction with noradrenaline. To perform the statistical analysis, the SigmaStat Statistical Analysis System program, version 3.5 (2006), was used, and by applying Student’s *t*-test, the statistical significance between the two groups was examined. The one-way ANOVA method was used to analyze comparisons between more than two groups, and the Dunnett post hoc test was utilized afterwards. Whenever normality and/or homoscedasticity criteria by the Kolmogorov–Smirnov test and Levene’s mean tests, respectively, were not met, a Kruskal–Wallis one-way analysis of variance on ranks, followed by Dunn’s method, was applied. The two-way ANOVA method was used to analyze comparisons among multiple groups with two independent variables and to determine a possible interaction between them, and the Holm–Sidak post hoc test was utilized afterwards. Significant differences were considered when the probability levels were below 5% (*p* < 0.05). The statistical data were graphically designed using Origin 2021 9.8.0.200 software.

## 5. Conclusions

In conclusion, our findings demonstrate that BPA (0.02 and 2 μM) exposure can affect the vasoactive response of SMC-MCAs, decreasing the vasocontractile response of NA and promoting a decrease in the vasorelaxant response of SNP. This effect of BPA was also obtained in the results of gene expression, where it was shown that the sGC protein and BK_Ca_ 1.1 α-subunit channels increased their expression, which is the main pathway of SMC relaxation. These results suggest an impairment of the SMC-MCA vasoactive response induced by intermediate BPA concentrations, an effect not attained for the lowest or highest exposure concentrations (showing a non-monotonic inverted U-shaped response). All these findings point to the possibility that BPA exposure modifies SMC-MCAs’ vascular homeostasis by interfering with the NO and sGC pathways and may, thus, be connected to the development of ischemic stroke episodes, which are the most common type of vascular disease and, as such, are a major concern for human health, as they rank among the world’s leading causes of death and disability. For this reason, additional research is required to provide a deeper understanding of this involvement and the associated mechanisms.

## Figures and Tables

**Table 1:** Statistical analysis of Figure 3B obtained by the two-way ANOVA method, followed by multiple comparisons vs. control group (Holm–Sidak method). The *p*-values (probability of significance) are shown only after 10 min of observation, when the statistical difference was observed.

BPA Concentrations	10 min	12 min	14 min	16 min	18 min	20 min
0.002 μM	0.090	0.114	**0.017**	**<0.001**	**<0.001**	**<0.001**
0.02 μM	**0.008**	**0.003**	**<0.001**	**<0.001**	**<0.001**	**<0.001**
0.2 μM	**0.002**	**<0.001**	**<0.001**	**<0.001**	**<0.001**	**<0.001**
2 μM	**0.006**	**0.001**	**<0.001**	**<0.001**	**<0.001**	**<0.001**
20 μM	**0.015**	**0.003**	**<0.001**	**<0.001**	**<0.001**	**<0.001**

**Table 2:** Primers utilized for qPCR.

Gene	Sequences of Primers (5′-3′)	Fragment Size (bp)	Annealing Temperature (°C)	GenBank Accession No.
Cyclophilin A	Fw: 5-CAA GAC TGA GTG GCT GGA TGG-3 Rv: 5-GCC CGC AAG TCA AAG AAA TTA GAG-3	163	60	NM_017101.1
BK_Ca_ 1.1 α	Fw: 5-ACT TCG CTT CAG GAC AAG GA-3 Rv: 5-CTG TCC ATT CCA GGA GGT GT-3	122	60	NM_031828.2
BK_Ca_-β1	Fw: 5-CCA GGA ATC CAC CTG TGA CT-3 Rv: 5-TCA CAT CAA CCA AGG CTG TC-3	218	60	NM_019273.1
Soluble guanyl cyclase 1 (sGC)	Fw: 5-CAA CAG TGT GGA GAG CTG GA-3 Rv: 5-ATC ATC TTC AGG GCC ATC AG-3	128	60	NM_017090.2
Protein kinase cGMP-dependent 1 (PKG1)	Fw: 5-CGT GAG GCT ATA CCG GAC AT-3 Rv: 5-GCA AAC GCT TCT ACC ACA CA-3	156	60	NM_001105731.4

## Data Availability

Data will be made available on request.

## Supplementary Materials

The following supporting information can be downloaded at: https://www.mdpi.com/article/10.3390/ijms26083896/s1.
